# Vertebrogenic Low Back Pain and Basivertebral Nerve Ablation: A Review of Mechanisms, Imaging-Driven Selection, and Clinical Outcomes

**DOI:** 10.3390/diagnostics16121943

**Published:** 2026-06-22

**Authors:** Daniele G. Romano, Ludovica Liguori, Giulia Pacella, Raffaele Natella, Federico Bruno, Francesco Arrigoni, Michela Bruno, Stefano Piemonte, Michele Fischetti, Mario Brunese, Marcello Zappia

**Affiliations:** 1 Department of Diagnostic and Interventional Neuroradiology, Ospedali Riuniti San Giovanni di Dio e Ruggi d’Aragona, 84131 Salerno, Italy; 2 Department of Medicine and Health Science “V. Tiberio”, University of Molise, 86100 Campobasso, Italy; 3 Fondazione Trotula de Ruggiero, 84121 Salerno, Italy; 4 Biomedical Research Centre, Gruppo Forte, 84124 Salerno, Italy; 5 Department of Biotechnological and Applied Clinical Sciences, University of L’Aquila, 67100 L’Aquila, Italy; 6 Interdisciplinary Department of Medicine, Section of Radiology and Radiation Oncology, University of Bari “Aldo Moro”, 70124 Bari, Italy; 7 Centro Diagnostico Ricerche Radiologiche s.r.l., Via Pierluigi da Palestrina 1, 70056 Molfetta, Italy; 8 Department of Precision Medicine, University of Campania “L.Vanvitelli”, 80138 Napoli, Italy

**Keywords:** basivertebral nerve ablation, vertebrogenic low back pain, Modic changes, chronic low back pain, radiofrequency ablation, Intracept, patient selection, spinal interventions

## Abstract

**Background:** Vertebrogenic low back pain (LBP) is a distinct subtype of chronic LBP (cLBP) arising from nociceptive sensitization of the basivertebral nerve (BVN) within pathologically altered vertebral endplates. Modic type 1 and type 2 changes on MRI are primary imaging biomarkers for patient selection. Basivertebral nerve ablation (BVNA), a minimally invasive intraosseous radiofrequency procedure, has emerged as a targeted treatment for this condition. This narrative review aims to synthesize current evidence on the pathophysiology of vertebrogenic LBP, patient selection criteria, procedural outcomes, safety profile, and cost-effectiveness of BVNA. **Methods:** We conducted this narrative review of the literature, encompassing randomized controlled trials (including the SMART and INTRACEPT studies), prospective registries, and real-world cohort studies evaluating BVNA for vertebrogenic LBP. Clinical and imaging-based selection criteria, procedural techniques, outcome measures, adverse events, opioid utilization, and healthcare utilization data were examined. **Results:** Evidence demonstrates consistent and durable reductions in pain and disability following BVNA, with a favorable safety profile. Complication rates are low, with vertebral compression fracture and procedure-related radicular pain reported as the most frequent adverse events. BVNA is associated with reduced opioid consumption and decreased overall healthcare utilization. Moreover, emerging data suggest efficacy beyond originally defined inclusion criteria, including cases of osteoporosis, multilevel Modic changes, adult spinal deformity, and complex comorbid presentations. **Conclusions:** BVNA represents an effective and safe treatment option within the multimodal management of vertebrogenic LBP. Current evidence supports a gradual expansion of procedural indications, with implications for healthcare resource optimization and opioid stewardship.

## 1. Introduction

CLBP is a worldwide leading cause of pain and disability [1]. Patients with vertebrogenic LBP typically present with a symptom profile marked by low sensitivity and specificity, frequently described as midline nociceptive pain exacerbated by sitting, forward flexion, and axial rotation, with symptoms becoming more pronounced under conditions of increased spinal loading [2].

Degenerative disk disease has traditionally been regarded as the primary explanation for a substantial proportion of chronic low back pain. Recent advances in histological, immunological, and radiological research have instead clarified a more specific pain generator: the vertebral endplates, whose pathological alterations give rise to vertebrogenic pain [3].

The vertebral endplates play an essential role in redistributing intradiskal pressures from adjacent vertebral bodies, helping to limit excessive mechanical stress and reducing disk bulging. The endplate is also the main route for nutrient diffusion into the largely avascular intervertebral disc, receiving its metabolic supply from the segmental spinal arteries that course through the endplate region [4].

Nociceptive nerve fibers of the basivertebral nerve are regarded as key contributors to pain generation, and histological analyses show an increased density of these fibers within the vertebral endplates when disk degeneration is present [5].

**Figure 1:**
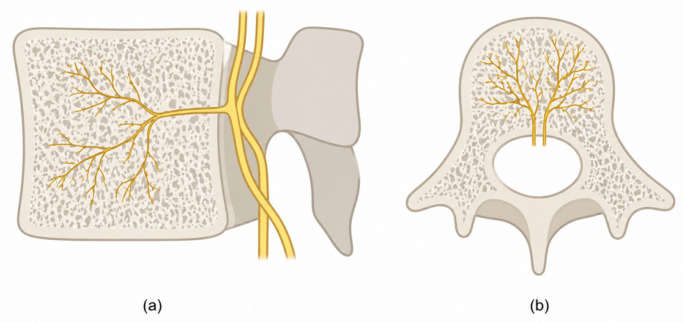
Anatomy of the basivertebral nerve. (**a**) Sagittal view. (**b**) Axial view.

The basivertebral nerve, typically appearing as a pair of nerve fibers, arises from the sinuvertebral nerves and enters the vertebral body posteriorly through the central vascular foramen. After entering, it travels through the central region of the vertebral body, generally accompanying the basivertebral vessels, and issues branches that extend toward the superior and inferior endplates [6]. An illustration of the BVN anatomy is provided in Figure 1.

**Figure 2:**
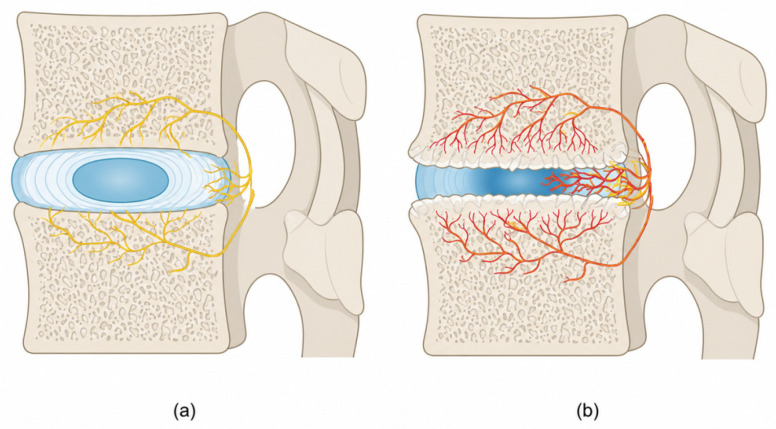
(**a**) Anatomy of BVN with a sinuvertebral nerve in the normal disk. (**b**) Pathological disk.

In normal conditions, the bony endplates contain numerous small free nerve endings, and when degeneration or inflammation occurs, these nociceptive structures are likely to become activated, giving rise to inflammatory pain (Figure 2).

Pathological alterations involving the basivertebral nerve endings and the bone marrow of the vertebral endplate give rise to characteristic findings detectable on magnetic resonance imaging (MRI), a non-invasive diagnostic tool that can also identify other potential causes of low back pain [2].

These alterations are collectively known as “Modic changes” [7] and are classified into three distinct types:

- Type 1—Subchondral bone marrow edema of the vertebral endplate, indicative of an inflammatory process, appearing on MRI as low signal intensity on T1-weighted sequences and high signal intensity on T2-weighted sequences.
- Type 2—Fatty degeneration/conversion of red hematopoietic marrow into yellow fatty marrow, likely secondary to marrow ischemia, appearing on MRI as high signal intensity on both T1- and T2-weighted sequences.
- Type 3—Bone remodeling with sclerotic tissue, characterized on MRI by low signal intensity on both T1- and T2-weighted sequences [8,9].

Modic type 1 and type 2 changes are considered highly specific for low back pain [1] and represent key criteria for selecting patients who may be candidates for basivertebral nerve ablation [10,11,12].

**Figure 3:**
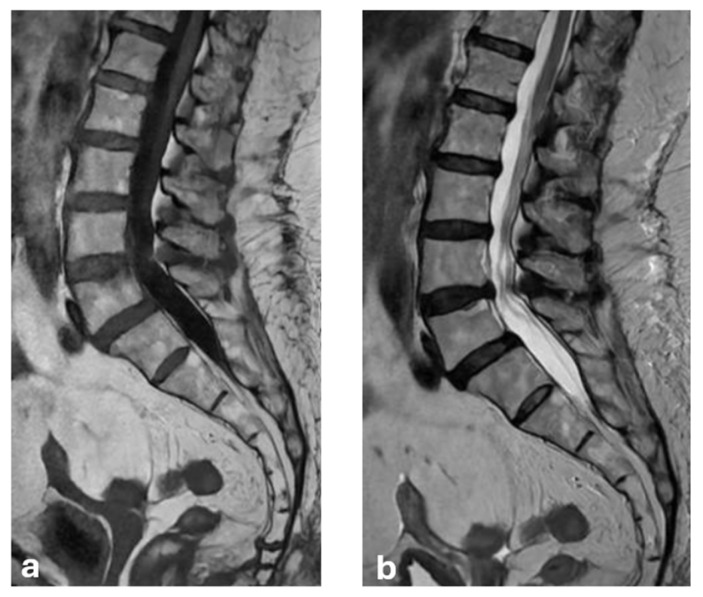
Type 1 Modic change. (**a**) T1-weighted. (**b**) T2-weighted.

**Figure 4:**
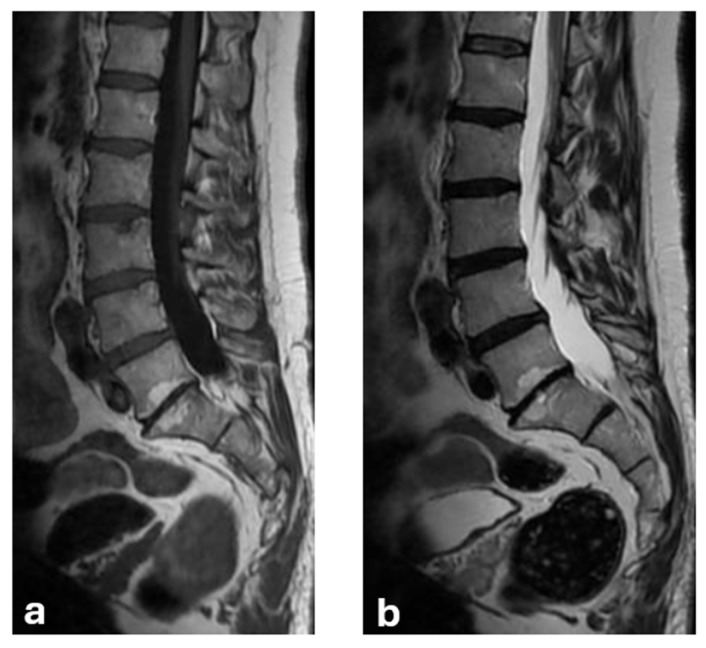
Type 2 Modic change. (**a**) T1-weighted. (**b**) T2-weighted.

Chronic low back pain employs a substantial socioeconomic burden globally, accounting for more years lived with disability than any other musculoskeletal condition [1,10]. It is estimated that up to 80% of the adult population will experience at least one episode of LBP during their lifetime, and a significant proportion will progress to chronicity, with consequent loss of productivity, increased healthcare utilization, and reduced quality of life [1]. Within this heterogeneous population, vertebrogenic LBP represents a phenotypically distinct subgroup that has historically been undertreated due to the absence of a clearly identifiable and targetable pain generator [2,3]. The recognition of the basivertebral nerve as a primary nociceptive driver, combined with the availability of reproducible MRI criteria for endplate pathology, has radically changed the diagnostic and therapeutic landscape for this subgroup [5,11]. This shift reflects the wide trend to precision pain medicine, in which imaging phenotyping is useful as a guide to the selection of targeted interventional therapies rather than empirical, symptom-based management [12]. In this context, BVNA emerges not only as an additional procedural option, but as the first intervention specifically designed to address the vertebrogenic pain mechanism at its anatomical source [13,14]. An example of Type 1 and of Type 2 changes is reported, respectively, in Figure 3 and Figure 4.

As reported by the International Society for the Advancement of Spine Surgery (ISASS), the prerequisites for considering BVNA are Modic type 1 or 2 changes, cLBP, and failure of non-surgical treatments for at least 6 months. In fact, this procedure has been incorporated since 2019 into the ISASS guidelines for LBP management as an effective treatment option in appropriately selected patients [13]. The various inclusion and exclusion criteria are analyzed in the review [13,14].

Currently, the only FDA-approved system for basivertebral nerve ablation is the Intracept device (Relievant Medsystems) [7,13].

This narrative review examines patient selection criteria, clinical outcomes, complication profile, and cost-effectiveness of BVNA, with particular attention to emerging evidence supporting its use beyond traditionally defined indications.

## 2. Methods

We prepared this narrative review in accordance with the Scale for the Assessment of Narrative Review Articles (SANRA), covering the justification of the topic, the aims of the review, the literature search, the referencing and the presentation of evidence levels and relevant endpoint data [15].

An updated literature search was performed on 9 June 2026 across two bibliographic databases, PubMed and Scopus, by using the following search strings:

PubMed: “basivertebral nerve”[Title/Abstract] AND (“ablation” OR “radiofrequency” OR “low back pain” OR “Modic”[Title/Abstract]).

Scopus: TITLE-ABS-KEY (“basivertebral nerve” AND (“ablation” OR “radiofrequency” OR “low back pain” OR “modic”)).

The search was restricted to only articles published in English. Reference lists of retrieved articles and relevant reviews were additionally screened individually to identify studies not captured by the electronic search (citation chaining).

The electronic search retrieved 100 records from PubMed and 114 from Scopus (total number = 214). After removal of duplicate records (automatic detection in EndNote, supplemented by manual screening) and exclusion of editorials, letters to the editor and their replies, and conference abstracts lacking original data, 124 final records were retained. Two authors independently screened these by title and abstract for relevance to the review objectives, with disagreements resolved by consensus; potentially eligible articles were then assessed by full text, and the most representative studies were selected for narrative synthesis. Studies were considered eligible if they reported clinical or imaging outcomes following BVNA in adult patients with chronic axial LBP and documented Modic type 1 or type 2 changes on MRI. Eligible study designs included randomized controlled trials, prospective registries, real-world cohort studies, systematic reviews, meta-analyses, and case reports. Studies focused on other interventional procedures for LBP without direct comparison to or inclusion of BVNA outcomes were excluded.

Consistent with the narrative methodology, no formal risk-of-bias or quality-assessment tool was applied; however, the level of evidence of each included study was explicitly considered when interpreting findings and drawing conclusions (Table 1). No statistical pooling of data was performed.

## 3. Results and Discussion

### 3.1. Patient Selection and Procedure

Chronic axial LBP is frequently multifactorial, and vertebrogenic pain commonly overlaps and coexists with other nociceptive sources like discogenic, facetogenic, and myofascial or sacroiliac pain. Vertebrogenic pain is mediated by the basivertebral nerve and shares many clinical features with discogenic pain (it is a predominantly midline axial pain, aggravated by sitting, forward flexion, and activity), whereas facetogenic pain is typically paramedian and exacerbated by extension; in practice these patterns frequently co-occur, and no single clinical feature is pathognomonic [1,2]. Modic type 1 and type 2 changes remain the most specific imaging biomarker for the vertebrogenic component [11], but their presence does not exclude concurrent discogenic or facetogenic contributors. From a practical standpoint, this phenotypic overlap has two implications: it underscores the importance of rigorous patient selection, including clinical correlation and exclusion of a dominant facetogenic, radicular, or stenotic source (the latter two being contraindications; Table 2), and it may account for partial or absent responses to BVNA in patients in whom vertebrogenic pain is only one of several coexisting pain generators.

Compared to traditional treatments for cLBP, in the literature BVNA has shown high response rates, particularly when in strict adherence to the established indications and contraindications (Table 2), which remain essential [13,19].

Assessment of symptomatic improvement has relied on validated patient-reported outcome measures, allowing for standardized comparison across studies. The most used instruments include:

- Oswestry Disability Index: A 10-item questionnaire, scored from 0 to 5, evaluating the interference degree of LBP in essential daily activities. The total score (0–50) is converted into a percentage (0–100%), with higher values indicating greater disability [20,32].
- Visual Analog Scale (VAS): A 100 mm horizontal line on which patients mark the point corresponding to their perceived pain intensity. The clinician then measures the distance in millimeters to obtain a score ranging from 0 to 100, providing a continuous measure of subjective pain severity [33].
- Beck Depression Inventory (BDI): Used to quantify the severity of depressive symptoms, which may influence pain perception and treatment response [19].

These standardized tools have been instrumental in demonstrating the clinical effectiveness of BVNA across diverse patient populations and study designs.

The procedure is performed with the patient in the prone position on the angiography table, using support to optimize lumbar extension and with the arms positioned above the head. After sterile field preparation and administration of preprocedural antibiotic prophylaxis (typically 2 g of intravenous cefazolin), the injection site is anesthetized with lidocaine. A transpedicular approach is then used to access the target vertebral body with the Intracept trocar. Once the posterior margin of the vertebral body is reached, the introducer is removed, and a J-stylet is advanced into the appropriate position within the posterior third of the vertebral body. Under fluoroscopic guidance, the radiofrequency probe is inserted, and ablation is performed for 15 min at 85 °C. At the end of the procedure, all instruments are removed, and a hemostatic patch is applied to the skin entry sites, followed by manual compression for approximately 5 min or until hemostasis is achieved [7,13].

### 3.2. Clinical Outcomes

The SMART trial by Fischgrund et al. [16] remains to date the largest multicenter, randomized, double-blind, sham-controlled study of BVNA. It includes 225 patients with chronic LBP (>6 months) refractory to conservative care, with ODI ≥ 30, VAS ≥ 4, and Modic type 1 or 2 changes on MRI. At three months, ODI and VAS improved in both the active and sham arms, reflecting a clinically meaningful non-specific response attributable to placebo effect, regression to the mean, and the natural history of LBP.

Therefore, the inference of a true treatment effect stands not on within-arm improvement but on the magnitude and durability of the between-arm difference: the active arm showed significantly greater benefit, sustained at 12 and 24 months [4], with open-label extension data suggesting durability to five years [21]. Nevertheless, a maintained sham comparator was not available beyond the early postoperative period; therefore, a residual contribution of expectation and natural history to the long-term within-arm improvement cannot be entirely excluded, so the durable between-arm separation at the controlled timepoints should be regarded as the principal evidence of a specific treatment effect.

The INTRACEPT [17] trial was a parallel-group, randomized, open-label study conducted across multiple U.S. centers to compare BVNA with standard care. A total of 140 patients were enrolled with inclusion criteria broadly similar to those of the SMART trial, although INTRACEPT permitted patients with prior lumbar surgery and spinal stenosis. At three months, the BVNA group demonstrated significantly greater improvements than the standard-care cohort. The superiority of BVNA persisted at 12 months [18], including among crossover patients who initially received standard care and subsequently elected to undergo BVNA.

Several studies have also explored whether pain location or exacerbating factors influence treatment response [34,35], although most patients describe predominantly midline axial pain. Additional imaging features have been investigated as potential predictors of vertebrogenic pain; however, Modic changes consistently remain the most reliable and clinically meaningful imaging criterion for identifying candidates likely to benefit from BVNA [34]. Taken together, within its label indication (chronic axial vertebrogenic LBP with concordant Modic type 1 or 2 changes between L3 and S1, refractory to ≥6 months of conservative care), BVNA is supported by Level 1–2 evidence: the two randomized controlled trials above (SMART and INTRACEPT), pooled analyses of the pivotal trials [20,21], a prospective real-world cohort [23], and a systematic review with single-arm meta-analysis rated as moderate-quality under GRADE [22].

However, the pivotal trials and most subsequent analyses were sponsored by (or conducted with investigator-initiated funding from) the device manufacturer Relievant Medsystems, now Boston Scientific, and the systematic review itself cautions that the predominance of industry-funded studies introduces a risk of bias, only partially mitigated by the concordant findings of the few independently conducted studies [22].

Beyond this label indication, a growing but lower-level literature has explored BVNA in populations historically excluded from the pivotal trials, including patients with significant comorbidities or atypical presentations previously considered unsuitable. This evidence consists predominantly of single case reports and small retrospective series (Level 3–5) and should be regarded as hypothesis-generating rather than as justification for expanding routine practice.

Initially, metabolic bone diseases were regarded as contraindications to BVNA; however, evidence from the literature [24,27] demonstrates that patients with osteoporosis, once provided they do not have fragility fractures, can still achieve meaningful clinical benefit. Notably, cohorts with higher mean age than those included in earlier trials showed significant improvement without postprocedural complications. These preliminary reports suggest that selected osteoporotic patients without fragility fractures may still benefit from the procedure; however, given the elevated vertebral compression fracture risk in this group, osteoporosis should be treated as a risk factor justifying individualized risk–benefit assessment and bone-health optimization, rather than as a routinely acceptable indication.

In a single case report, Patel et al. [28] described multilevel BVNA (L3–S1) in an elderly patient with multilevel Modic changes and atypical features including unilateral radicular pain, with improvement in pain, mobility, and analgesic use (Level 5 evidence). A retrospective community series in adult spinal deformity [25] reported symptomatic benefit in patients with comorbidities such as spinal stenosis or spondylolisthesis; however, this same series documented a clinically relevant rate of postprocedural vertebral compression fractures in older, osteoporotic patients, underscoring that benefits must be weighed against procedural risk in this population.

Moreover, the case report by Gupta A. et al. [29] describes the first documented use of BVNA in a 50-year-old patient with ankylosing spondylitis and a 20-year history of chronic low back pain refractory to DMARD therapy. Following imaging confirmation of Modic changes at L2–L3, the patient underwent BVNA at 85 °C for 15 min and subsequently reported sustained symptom relief without adverse effects. As an isolated case report (Level 5 evidence), this observation is hypothesis-generating only and does not establish efficacy of BVNA in inflammatory autoimmune spondyloarthropathies; controlled studies should be conducted before any clinical recommendation.

Furthermore, for patients with mixed pain phenotypes not responding to conservative treatments such as physical therapy, pharmacologic management, or facet joint blocks, a sequential approach combining surgical decompression (e.g., laminectomy) followed by BVNA has demonstrated clinical benefit. This strategy appears effective in addressing both radicular symptoms and vertebrogenic axial pain [30]. The study by Odonkor C.A. et al. further supports the versatility of BVNA, showing meaningful improvement in patients with scoliosis undergoing Schroth therapy [26]. Notably, the combined-treatment arm exhibited significant reductions in pain (VAS), disability [36], opioid consumption, and overall healthcare utilization as early as 12 months. Both the staged-decompression case report [30] (Level 5) and the retrospective propensity-matched scoliosis cohort [26] (Level 3) findings are preliminary; despite the encouraging key findings, they require prospective, adequately powered validation before BVNA can be recommended in these complex presentations.

### 3.3. Complications

Although basivertebral nerve ablation (BVNA) is generally considered a safe and well-tolerated procedure, several complications have been reported. Vertebral compression fractures (VCFs) are the most frequently described adverse event. Although in large real-world and registry data the overall incidence is low, approximately 1.55% [31], it conceals a higher risk in vulnerable subgroups. In a retrospective community series of adult spinal deformity patients [25], postprocedural VCF occurred in approximately 10% (9/77) of the higher-comorbidity subgroup, who were predominantly elderly (mean age ≈ 78 years) and osteoporotic. Therefore, reduced bone mineral density, advanced age, and multilevel treatment represent clinically meaningful risk factors rather than negligible ones. Accordingly, osteoporosis should not be regarded as a routine green light for BVNA: while it need not constitute an absolute contraindication, candidacy in osteoporotic or elderly patients requires individualized risk–benefit assessment, bone-health evaluation and optimization (e.g., DXA), exclusion of patients with prior fragility fractures, and explicit counseling regarding the elevated fracture risk [24,25,27].

A rare but documented complication is the development of postprocedural extradural hematoma, reported in a single case [37] and likely attributable to bleeding from the basivertebral venous plexus traversing the foramen. This risk can be mitigated through careful preprocedural evaluation of individual bleeding risk, including anticoagulant use and other hematologic factors.

The most common complication following BVNA is radicular pain at the treated level. This event is largely related to vertebral anatomy, particularly at L5 level, where the convex vertebral body shape may predispose to pedicle breach during transpedicular access. To minimize this risk, preprocedural MRI or CT should be used to calculate the optimal entry point, applying a validated formula that determines the necessary degree of ventral advancement before introducing the curved stylet [38], thereby avoiding premature medial angulation.

Overall, meticulous procedural planning, accurate imaging-based trajectory assessment, and appropriate patient selection substantially reduce the likelihood of complications. When performed in appropriately selected patients, including evaluation of bone health, BVNA maintains a favorable safety profile for vertebrogenic low back pain.

### 3.4. Cost-Effectiveness

As LBP represents a leading driver of global healthcare expenditure and remains a major cause of disability, BVNA has emerged as a safe and effective intervention capable not only of improving clinical outcomes but also of substantially reducing healthcare utilization [10]. Indeed, current evidence indicates that BVNA is associated with decreased reliance on pharmacologic therapies, particularly opioid analgesics [39], and provides durable clinical benefit extending up to 5 years [21].

In the evaluation of the risk–benefit profile of BVNA, radiation exposure also has to be considered, as the procedure is fluoroscopy-guided. In a single-center series of 55 patients, the mean fluoroscopy time was approximately 2.5 min (152.5 ± 84.3 s) and the mean cumulative radiation dose was 70.3 ± 53.0 mGy [40], values comparable to those reported for other fluoroscopically guided spinal and orthopedic procedures. Several factors increase fluoroscopy time and cumulative dose, including treatment of multilevel Modic changes, involvement of the S1 level, where sacroiliac anatomy complicates access, elevated BMI, and limited operator experience. Nevertheless, exposure can be mitigated through established dose-reduction strategies, such as pulsed and low-dose fluoroscopy, tight collimation, last-image-hold, maximizing operator distance and shielding, and CT- or navigation-guided targeting, and through accurate preprocedural trajectory planning that reduces repositioning attempts. Cumulative dose should be documented and monitored, particularly in patients requiring multilevel or repeat procedures.

With the explicit aim of expanding the safety margin of procedures, protocol modifications have also been explored. A lower-energy protocol of 7 min ablation at 75 °C versus the standard 15 min ablation at 85 °C [41] delivers less cumulative thermal energy and this may reduce thermal spread beyond the intended target, thereby theoretically lowering the risk of collateral thermal injury and of structural weakening of the vertebral body. This aim is particularly relevant in elderly or osteoporotic patients at higher baseline fracture risk. However, this potential safety advantage must be balanced with efficacy: the lower-energy protocol significantly reduced pain but yielded a lower proportion of clinical responders than the standard protocol. Direct comparative data on procedure-related complications between the two protocols are not yet available; therefore, the choice of protocol should be detected as a risk–benefit trade-off, considering the lower-energy settings a reasonable option when structural safety is the priority, pending dedicated comparative safety studies.

From a quantitative standpoint, a model-based cost-effectiveness analysis from a US payer perspective, anchored on the INTRACEPT trial, estimated an incremental cost-effectiveness ratio of approximately US$11,400 per quality-adjusted life-year (QALY) gained over a 5-year horizon, well below the conventional willingness-to-pay threshold of US$100,000–150,000/QALY, with a >99% probability of being cost-effective [42].

It should be noted, however, that this analysis was sponsored by the device manufacturer, and independent economic evaluations remain lacking. Considering a broader health economics perspective, BVNA compares favorably with alternative interventional strategies for cLBP. Spinal cord stimulation, while effective in selected neuropathic pain presentations, carries significantly higher upfront device costs and a non-negligible rate of hardware-related complications requiring revision. Lumbar fusion surgery, often considered in patients with structural instability, is associated with substantially greater perioperative risk, longer rehabilitation periods, and higher rates of adjacent segment pathology over time. In contrast, BVNA is an outpatient procedure with a short recovery period, low complication rate, and durable analgesic effect, rendering it cost-effective across multiple healthcare systems and reimbursement models [14]. A further economic advantage lies in its opioid-sparing effect: reduced long-term opioid consumption not only lowers direct pharmaceutical costs but also diminishes the indirect costs associated with opioid-related adverse events, dependency, and healthcare utilization [39]. As value-based care models increasingly prioritize outcomes per unit cost, the profile of BVNA, combining procedural simplicity, reproducible efficacy, and downstream resource savings, positions it as an economically sustainable option within the multimodal management of vertebrogenic LBP [10,14].

### 3.5. Future Therapies

Looking ahead, several emerging therapeutic strategies may further expand the management options for vertebrogenic and mixed-etiology low back pain.

Beyond conventional MRI, functional imaging is being explored to refine candidate selection. In a retrospective study of 52 patients, SPECT/CT showed substantial concordance with Modic changes, particularly type 1, suggesting that metabolically active endplate uptake may complement MRI, or serve as an alternative when MRI is contraindicated or inconclusive, in identifying symptomatic vertebrogenic levels [43]. These findings are preliminary and retrospective, and prospective correlation with treatment outcomes is needed.

The study by Sayed D. et al. [44] evaluated the feasibility of a novel multitined expandable electrode (Subsidio™) for BVNA. Through a series of simulations performed on lumbosacral CT reconstructions, the authors demonstrated that the device can be accurately positioned within the vertebral body while accommodating individual anatomical variability. By assessing multiple spatial parameters, including tip-to-tip distances, cannula trajectories, and angulation, the study provides foundational evidence supporting the potential adoption of alternative electrode designs capable of achieving effective ablation of the basivertebral nerve plexus.

Finally, the investigation by Kim H.S. et al. [7] assessed the safety and efficacy of the minimally invasive TEBLA technique, which utilizes a 1414 nm Nd:YAG laser. Although conducted in a relatively small cohort of 14 patients, the study showed that TEBLA, performed via a transforaminal epiduroscopic approach and allowing direct visualization and intraoperative provocation, resulted in substantial pain reduction without any reported complications. These preliminary findings suggest that laser-based epiduroscopic BVN ablation may represent a promising adjunct or alternative to conventional radiofrequency-based techniques.

## 4. Conclusions

BVNA has evolved into a reproducible intervention with consistent evidence of safety and efficacy when performed on appropriately selected patients. Randomized data from the SMART and INTRACEPT trials, supported by real-world cohorts, establish its role in chronic vertebrogenic LBP with concordant Modic type 1 or 2 changes refractory to conservative care.

However, several caveats qualify a noncritical appraisal of the existing evidence. The pivotal randomized trials and most of the subsequent pooled analyses were sponsored by the sole FDA-approved device manufacturer (Relievant Medsystems, now Boston Scientific), and even the available systematic review was conducted under an investigator-initiated grant from the same company. Genuinely independent data remain limited. This concentration of industry funding carries a risk of publication and reporting bias, only partly alleviated by the concordant results of the few independently conducted studies. Independent, adequately powered studies and prospective multicenter registries are therefore needed to corroborate the efficacy, safety, and cost-effectiveness estimates reported to date.

Furthermore, evidence supporting the expansion of indications also to osteoporosis, spinal deformity, or inflammatory conditions remains largely anecdotal, with case reports and small series that generate hypotheses rather than support practice change. Outside trial settings, long-term durability beyond five years and effectiveness in the heterogeneous patients encountered are still incompletely characterized.

Therefore, the most meaningful aim is not a further confirmation of efficacy in ideal candidates but a rigorous prospective evaluation in broader populations, with independent funding, standardized outcome measures, and follow-up extending beyond current benchmarks.

The central challenge remains the refining of the imaging-to-patient pathway, identifying within real-world populations those most likely to benefit from treatment. This phenotype-driven perspective aligns with recent integrative frameworks linking MRI phenotyping to image-guided intervention selection in chronic low back pain [12].

From a translational standpoint, the integration of quantitative MRI biomarkers—including endplate signal area, Modic change grading, and disk height indices—into standardized patient selection algorithms represents a priority for future research [12]. Artificial intelligence-assisted image analysis may further refine candidate identification by reducing operator-dependent variability in Modic change assessment and enabling automated extraction of relevant morphological features from large imaging datasets [8]. Parallel advances in procedural technology, including novel electrode geometries and real-time temperature monitoring, may enhance ablation precision and expand the anatomical range of treatable levels [44]. Multicenter registries with prospective data collection and standardized reporting of outcomes, adverse events, and patient characteristics are needed to generate the level of evidence required for broader guideline endorsement and payer acceptance [14,22].

When these conditions are met, BVNA represents a valuable and increasingly applicable tool in the multimodal management of vertebrogenic pain, whose full potential is still being defined.

## Figures and Tables

**Table 1:** Summary of key included studies.

Study (REF)	Design	n	Follow-Up	Primary Outcome(s)	Funding/Sponsor	LOE
**Fischgrund/Koreckij—Smart [3,4,16]**	RCT, double-blind, sham-controlled	225	Up to 24 mo	ODI, VAS	Industry—Relievant Medsystems (now Boston Scientific)	1
**Khalil/Smuck—intracept [17,18]**	RCT, open-label vs. standard care	140	12 mo (+crossover)	ODI, VAS, responder rate	Industry—Relievant Medsystems	1–2
**Macadaeg [19]**	Prospective, single-arm, open-label (community)	48 enrolled (45 completed)	12 mo	ODI, VAS (+ EQ-5D-5L, SF-36)	Industry—Relievant Medsystems	2
**Boody [20]**	Pooled cohort, 3 prospective trials, 33 centers	296	Varies	Predictors of treatment success	Industry—Relievant Medsystems	2
**Khalil [21]**	5 yr pooled analysis, 3 prospective trials	249/320 completed (mean 5.6 yr)	5 yr	ODI, NPS; safety	Industry—Relievant Medsystems	2
**Conger [22]**	Systematic review + single-arm meta-analysis (GRADE moderate)	414 (6 populations)	6 & 12 mo	≥50% pain relief; ≥15 pt ODI	Investigator-initiated grant, Relievant (independent conduct)	1–2
**Mccormick [23]**	Prospective real-world cohort, single-arm	60	3 & 12 mo	ODI improvement	Academic/investigator-initiated (Utah)	2
**Bellow [24]**	Real-world (osteoporosis/osteopenia)	—	—	Safety, effectiveness		3–4
**Fogel [25]**	Retrospective case series, ASD (community)	118 (Grp B 77)	Last FU	VAS, ODI; **VCF 9/77 ≈ 10% (elderly, osteoporotic)**	Community practice; no industry funding stated	4
**Odonkor [26]**	Retrospective, propensity-matched cohort, multicenter	76 (44 matched, 22/grp)	12 mo	≥15-pt ODI; opioid, healthcare use	Academic (Yale)	3
**Lopez [27]**	Case report (advanced osteopenia)	1	—	Pain, function	None (case report)	5
**Patel [28]**	Case report (elderly, multilevel Modic)	1	—	Pain, mobility	None (case report)	5
**Gupta [29]**	Case report (ankylosing spondylitis)	1	—	Symptom relief	None (case report)	5
**Faughender [30]**	Case report (staged decompression + BVNA)	1	—	Axial + radicular pain	None (case report)	5
**Chau [31]**	Retrospective, national database	—	—	VCF incidence (≈1.55%)		3
**Kim—Tebla [7]**	Preliminary open-label (laser)	14	—	Pain		4

Levels of evidence are graded according to the Oxford Centre for Evidence-Based Medicine (OCEBM) criteria (1 = high-quality randomized controlled trial; 5 = case report or mechanism-based reasoning). SMART and INTRACEPT denote the two pivotal randomized trials. Cells marked “to confirm/verify” indicate data not retrievable from indexed sources, to be completed from the primary publication. Abbreviations: ASD, adult spinal deformity; BVNA, basivertebral nerve ablation; EQ-5D-5L, EuroQol 5-Dimension 5-Level questionnaire; FU, follow-up; GRADE, Grading of Recommendations, Assessment, Development and Evaluation; grp, group; LoE, level of evidence; mo, months; n, number of participants; NPS, numeric pain score; ODI, Oswestry Disability Index; RCT, randomized controlled trial; SF-36, 36-Item Short-Form Health Survey; Tebla, transforaminal epiduroscopic basivertebral nerve laser ablation; VAS, visual analog scale; VCF, vertebral compression fracture.

**Table 2:** Indications and contraindications for basivertebral nerve ablation.

**Indications**	• Chronic axial low back pain lasting > 6 months • Refractory to conservative therapy for at least 6 months • Modic type 1 or type 2 changes on MRI at one or more levels between L3 and S1 • ODI ≥ 30/100 • VAS ≥ 4/10
**Contraindications**	• Chronic low back pain of <6 months’ duration • Radicular pain or neurological deficit in a dermatomal distribution • Previous lumbar spine surgery • Symptomatic spinal stenosis (including neurogenic claudication) • Metabolic bone disease with a history of vertebral fragility fractures • Radiographic evidence of an alternative pain etiology • Disk protrusion or extrusion > 5 mm • Spondylolisthesis > 2 mm at any level • Spondylolysis at any level • Modic changes at levels outside L3–S1 • Ablation zone located < 10 mm from the spinal canal • Facet arthropathy or facet edema associated with axial low back pain • Use of long-acting narcotics • Active spinal or systemic infection • Bleeding diathesis • BDI > 24 or three or more Waddell signs • BMI > 40 • Severe cardiac or pulmonary impairment • Contraindications to MRI (pacemakers, defibrillators, active implanted devices, pregnancy, breastfeeding)

## Data Availability

No new data were created or analyzed in this study. Data sharing is not applicable to this article.

## Abbreviations

The following abbreviations are used in this manuscript:

BDI	Beck Depression Inventory
BMI	Body Mass Index
BVN	Basivertebral Nerve
BVNA	Basivertebral Nerve Ablation
cLBP	Chronic Low Back Pain
CT	Computed Tomography
DMARD	Disease-Modifying Antirheumatic Drug
FDA	Food and Drug Administration
ISASS	International Society for the Advancement of Spine Surgery
LBP	Low Back Pain
MRI	Magnetic Resonance Imaging
Nd:YAG	Neodymium-doped Yttrium Aluminum Garnet
ODI	Oswestry Disability Index
RCT	Randomized Controlled Trial
TEBLA	Transforaminal Epiduroscopic Basivertebral Laser Ablation
VAS	Visual Analog Scale
